# Desensitization of Brentuximab Vedotin in a Patient with Hodgkin Lymphoma

**DOI:** 10.7759/cureus.2981

**Published:** 2018-07-13

**Authors:** Sidra Khalid, Aariez Khalid, Bernadette A Clark, Hamed Daw

**Affiliations:** 1 Internal Medicine/Residency, Fairview Hospital/Cleveland Clinic, Cleveland, USA; 2 Biomedical Science, University of Guelph, Guelph, CAN; 3 Oncology, Fairview Hospital/Cleveland Clinic, Cleveland, USA; 4 Hematology and Oncology, Fairview Hospital/Cleveland Clinic, Cleveland, USA

**Keywords:** brentuximab vedotin, hypersensitivity, desensitization, hodgkin lymphoma

## Abstract

Brentuximab vedotin is a monoclonal antibody that targets the CD30 antigen. It is indicated for the treatment of Hodgkin lymphoma. Hypersensitivity reactions have occurred during infusions of brentuximab vedotin, ranging from mild to severe. We report a case of a 46-year-old male with stage IV nodular sclerosis Hodgkin lymphoma who developed a hypersensitivity reaction to brentuximab vedotin. He experienced a generalized rash, facial swelling, and mild airway obstruction. In order to continue treatment with brentuximab vedotin, we implemented a desensitization protocol. He was premedicated and a 12-step process was performed in which brentuximab vedotin was titrated over three hours. The protocol was successful, allowing the patient to receive subsequent infusions without hypersensitivity reactions.

## Introduction

Brentuximab vedotin is a monoclonal antibody against the CD30 antigen present on Reed-Sternberg cells [1]. Brentuximab vedotin is indicated for the treatment of Hodgkin lymphoma in previously untreated advanced disease, relapsed or refractory disease and for consolidation after autologous hematopoetic stem cell transplantation. One of the adverse effects of brentuximab vedotin is hypersensitivity reaction during infusion. In clinical trials using brentuximab vedotin as monotherapy, 13% of patients experienced an infusion reaction, out of which 1.2% of patients experienced a grade 3 infusion reaction. When brentuximab vedotin was studied in combination with chemotherapy, 9% of patients experienced an infusion reaction, out of which 0.4% experienced grade 3 reactions. The hypersensitivity reaction can range from a mild infusion reaction to a more severe reaction including anaphylaxis. Although the manufacturer’s information on brentuximab vedotin recommends permanently discontinuing brentuximab vedotin if an anaphylactic reaction occurs, several case reports have shown success with a desensitization protocol [2]. In this case, we report a successful desensitization regimen with brentuximab vedotin, with no further hypersensitivity reactions noted during the subsequent infusions.

## Case presentation

**Figure 1:**
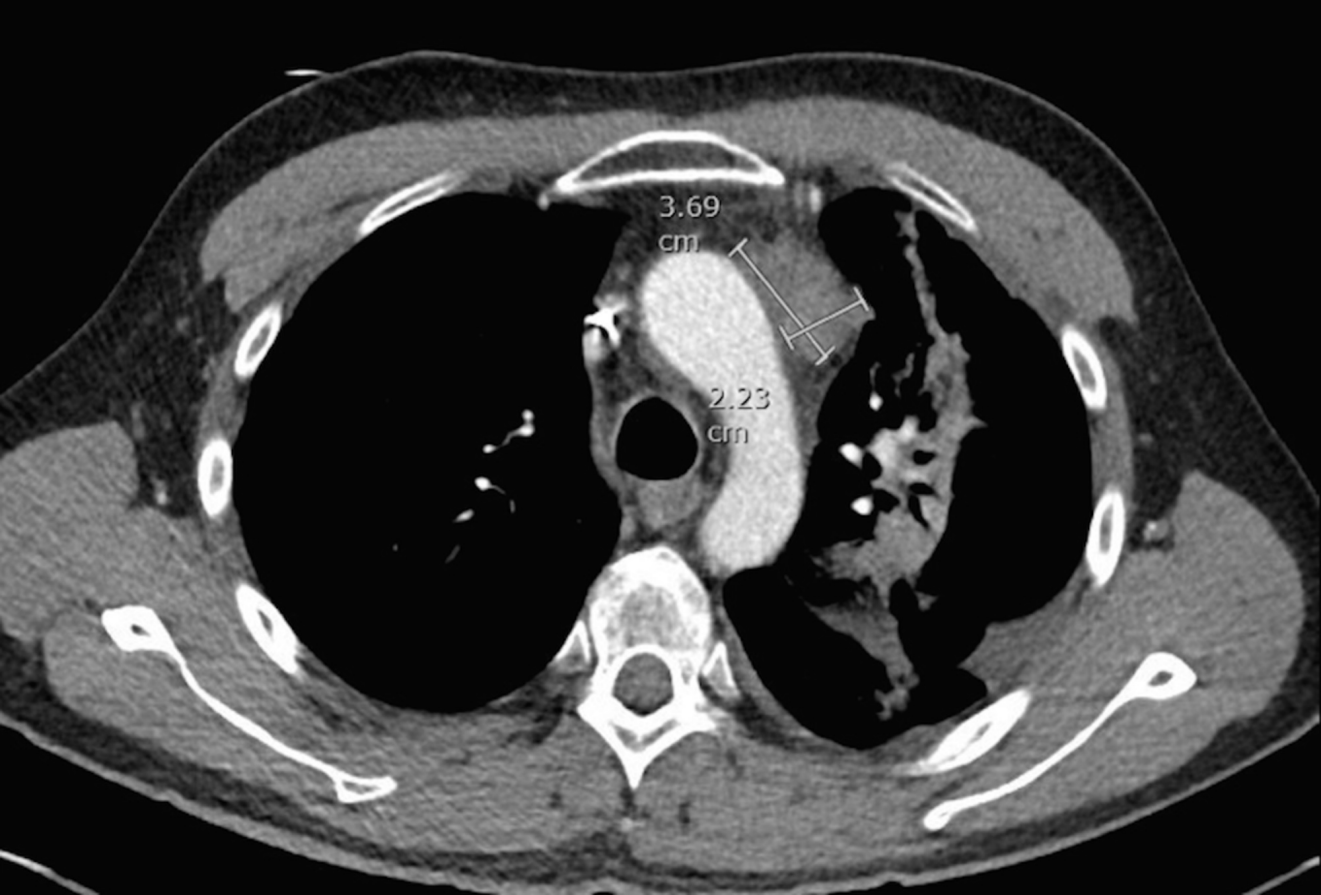
Computed tomography (CT) scan of the chest showing the anterior mediastinal mass

**Figure 2:**
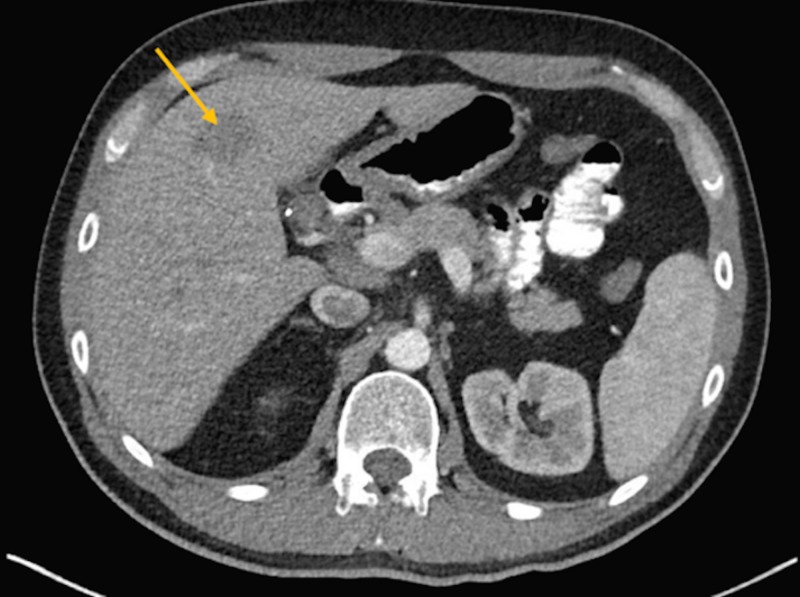
Computed tomography (CT) scan of the liver showing a mass in the left lobe

A 46-year-old male with stage IVB nodular sclerosis Hodgkin lymphoma received treatment with six cycles of ABVD (doxorubicin, bleomycin, vinblastine, dacarbazine) and then autologous stem cell transplant. Despite these treatments, he had progression of his disease. The positron emission tomography (PET) showed hypermetabolic activity in the anterior mediastinal mass and left hilar lymph node. He underwent radiation to the mediastinum. A subsequent computed tomography (CT) scan of the chest and liver showed a progression of the anterior mediastinal mass measuring 3.7 x 2.2 cm and a new mass in the left lobe of the liver measuring 2.9 x 2.8 cm (Figures 1-2).

A liver biopsy of the mass showed recurrent classical Hodgkin lymphoma. He was started on brentuximab vedotin at a dose of 1.8 mg/kg after administration of oral diphenhydramine 25 mg and oral acetaminophen 650 mg. Three weeks later, the second cycle was administered at the same dose with the same premedications. After receiving 10 mL of the medication, he developed facial flushing, swelling, generalized rash, and a scratchy sensation in his throat. He was given hydrocortisone 100 mg intravenously and his symptoms improved. Prior to the next cycle of treatment, the diphenhydramine 25 mg was changed to the intravenous (iv) route and dexamethasone sodium phosphate 20 mg iv and famotidine 20mg iv were added. During his third brentuximab vedotin infusion, he developed facial flushing and swelling, scratchy throat, and a rash. The infusion was stopped and he was given diphenhydramine 25 mg iv. His symptoms resolved gradually. It was decided to admit him to the hospital for cycle four for brentuximab vedotin desensitization. He was premedicated with methylprednisolone 60 mg iv, acetaminophen 650 mg orally, famotidine 20 mg iv, diphenhydramine 50 mg iv, and montelukast 10 mg orally. The following protocol was used for brentuximab vedotin at 1.8 mg/kg (Table 1).

**Table 1:** Twelve-step desensitization protocol for brentuximab vedotin

Brentuximab 1.8 mg/kg = 149 mg total dose		
Elapsed Timed	Administration Rate	Ratio of total dose
Bag 1: 100 fold dilution made as 1.49 mg in 250 mL. Total dose infused from Bag 1 = 9.25 mL x (1.49 mg/250 mL) = 0.05513 mg		
0 minutes	2 mL/hr	1:50,000
15 minutes	5 mL/hr	1:20,000
30 minutes	10 mL/hr	1:10,000
45 minutes	20 mL/hr	1:5,000
Bag 2: 10 fold dilution made as 14.9 mg in 250 mL. Total dose infused from Bag 1 = 18.75 mL x (14.9 mg/250 mL) = 1.1175 mg		
75 minutes	5 mL/hr	1:2,000
90 minutes	10 mL/hr	1:1,000
105 minutes	20 mL/hr	1:500
120 minutes	40 mL/hr	1:250
Bag 3: Remainder of dose made as 147.8 mg in 250 mL		
135 minutes	10 mL/hr	1:100
150 minutes	20 mL/hr	1:50
165 minutes	40 mL/hr	1:25
180 minutes	80 mL/hr	Infuse remainder of dose (137mg) at 80mL/hr

The patient had no complications during the desensitization process. For cycle five, he was admitted as well, and similarly, had no hypersensitivity reactions. The dose of brentuximab vedotin was reduced to 1.2 mg/kg on cycle six for worsening neuropathy, but the desensitization was continued. After cycle six, the treatment with brentuximab vedotin was discontinued due to worsening neuropathy and progression of the cancer. The PET scan showed several new hypermetabolic foci in the liver with mildly hypermetabolic lung nodules slightly progressed from prior imaging studies. He was started on nivolumab for further management of his Hodgkin lymphoma.

## Discussion

Based on the American Cancer Society, in 2018, there are about 8500 new cases of Hodgkin lymphoma. There are about 1050 deaths that occur due to this disease. It is commonly diagnosed in the third decade of life [3]. According to the World Health Organization (WHO), Hodgkin lymphoma is classified as nodular lymphocyte predominant or classical. The classical form is further subdivided into nodular sclerosis, lymphocyte-rich, mixed cellularity, lymphocyte-depleted [4]. Nodular sclerosis classical Hodgkin lymphoma is the most common subtype accounting for about seven out of every ten cases. The Ann Arbor classification system is used for staging Hodgkin lymphoma. Stage I – involvement of a single lymphatic site to localized involvement of a single extralymphatic organ (except lymph nodes); stage II – involvement of two or more lymph node regions on the same side of the diaphragm, or localized involvement of a single extralymphatic organ site with associated regional lymph nodes; stage III – involvement of lymph node regions on both sides of the diaphragm, with extension to other adjacent lymph nodes and/or spleen; stage IV – diffuse involvement of one or more extralymphatic organs with or without lymph node involvement, or involvement of liver, bone marrow, cerebrospinal fluid, or isolated extralymphatic organ with or without lymph node involvement but involving a distant site. These stages can be further sub classified into A (no symptoms) and B (symptoms of unexplained weight loss >10% in six months, fever above 38°C, night sweats). Clinical staging is performed by CT thoracic, abdomen and pelvis with or without PET scan [1].

Treatment is based on the stage of the disease. For stages IA or IIA, chemotherapy, combined chemotherapy, and radiation therapy, or radiation therapy alone are options. Treatment options could include ABVD for four to six cycles; ABVD for two cycles plus radiation therapy (20 Gy or 30 Gy); or radiation therapy alone. For stage III or IV disease, bulky disease (>10 cm mass or mediastinal disease involving >33% of the transthoracic diameter) or B symptoms; combination chemotherapy with or without radiation therapy is recommended. ABVD for six to eight cycles is the standard of care [1]. 

In relapsed Hodgkin lymphoma, patients can undergo re-induction with the same chemotherapy or another chemotherapy regimen followed by autologous hematopoietic stem cell transplant. Subsequently after stem cell transplant, if there is a relapse, an involved-field radiation therapy for residual masses could be performed [1]. Another option is brentuximab vedotin, which is a monoclonal antibody which targets the CD30 antigen on the Reed-Sternberg cells [5]. It is FDA (US Food and Drug Administration) approved for patients who have relapsed after autologous stem cell transplantation or have failed at least two lines of therapy. On March 20, 2018, the FDA approved brentuximab vedotin to treat adult patients with previously untreated stage II or IV classical Hodgkin lymphoma in combination with chemotherapy based on results from the ECHELON-1 trial [6]. As brentuximab vedotin is an important treatment option for patients with Hodgkin lymphoma, it is crucial to know about its hypersensitivity reaction and its plausible method of desensitization. Our case describes a hypersensitivity reaction to brentuximab vedotin, which manifested as a rash and mild airway obstruction. Based on case reports about brentuximab vedotin desensitization, we implemented the protocol in our patient (Table 2).

 

**Table 2:** Case reports of desensitization protocols for brentuximab vedotin

Case Reports	Hypersensitivity Reaction	Premedication Agents Used	Desensitization Process
Arora et al. [7]	Anaphylaxis	Methylprednisolone 125 mg Diphenhydramine 50 mg Ranitidine 50 mg Montelukast 10 mg	Three brentuximab vedotin (1.8 mg/kg) dilutions of increasing concentrations administered at increasing rates over a 12-step process
DeVita et al. [8]	Anaphylaxis	Methylprednisolone 60 mg Diphenhydramine 50 mg Famotidine 20 mg Montelukast 10 mg Acetaminophen	Three brentuximab vedotin (1.8 mg/kg) dilutions of increasing concentrations were delivered at four increasing rates, over 13 consecutive steps
Fizesan et al. [9]	Anaphylaxis	Aspirin 500 mg Cetirizine 10 mg Montelukast 10 mg Ranitidine 150 mg	A 17-step protocol in which four brentuximab vedotin (1.8 mg/kg) dilutions were given at increasing concentrations This resulted in a hypersensitivity reaction, methylprednisolone 120 mg and dexachlorpheniramine 5 mg were given and added to future regimens (next protocol with 1 mg/kg, then 1.5 mg/kg, followed by 1.8 mg/kg)
O’Connell et al. [10]	Anaphylaxis	Four days of premedication with dexamethasone 4 mg/day Diphenhydramine 50 mg Famotidine 20 mg Cetirizine 10 mg Acetaminophen 1 g	Three brentuximab vedotin (1.8 mg/kg) dilutions of increasing concentrations administered at increasing rates over a 12-step process
Story et al. [11]	Hives	Methylprednisolone 60 mg Fexofenadine 180 mg	A 13-step protocol with 150 mg of brentuximab vedotin (1.8 mg/kg) administered at increasing concentrations

We implemented the desensitization protocol and premedicated our patient with 50 mg iv diphenhydramine, 20 mg iv famotidine, 60 mg iv methylprednisolone, 10 mg oral montelukast, and acetaminophen. We then followed the 12-step desensitization protocol, in which the concentration of the drug was gradually increased. Our patient underwent successful brentuximab vedotin desensitization and was able to undergo further treatment. Routinely, when there is disease progression with brentuximab vedotin then the next step would be to start treatment with anti-program cell death (PD)1 inhibitor, such as nivolumab.

## Conclusions

Through our case, we have highlighted that brentuximab vedotin is an important treatment option for patients with advanced Hodgkin lymphoma. Hypersensitivity reactions can occur with this drug, which can be successfully managed with desensitization.
